# The Mighty NUMT: Mitochondrial DNA Flexing Its Code in the Nuclear Genome

**DOI:** 10.3390/biom13050753

**Published:** 2023-04-27

**Authors:** Liying Xue, Jesse D. Moreira, Karan K. Smith, Jessica L. Fetterman

**Affiliations:** 1 Evans Department of Medicine, Boston University Chobanian & Avedisian School of Medicine, Boston, MA 02118, USA; 2 Department of Health Sciences, Programs in Human Physiology, Boston University Sargent College, Boston, MA 02215, USA

**Keywords:** nuclear-mitochondrial DNA segments  mitochondrial genome, mitochondrial DNA, bioinformatics

## Abstract

Nuclear-mitochondrial DNA segments (NUMTs) are mitochondrial DNA (mtDNA) fragments that have been inserted into the nuclear genome. Some NUMTs are common within the human population but most NUMTs are rare and specific to individuals. NUMTs range in size from 24 base pairs to encompassing nearly the entire mtDNA and are found throughout the nuclear genome. Emerging evidence suggests that the formation of NUMTs is an ongoing process in humans. NUMTs contaminate sequencing results of the mtDNA by introducing false positive variants, particularly heteroplasmic variants present at a low variant allele frequency (VAF). In our review, we discuss the prevalence of NUMTs in the human population, the potential mechanisms of de novo NUMT insertion via DNA repair mechanisms, and provide an overview of the existing approaches for minimizing NUMT contamination. Apart from filtering known NUMTs, both wet lab-based and computational methods can be used to minimize the contamination of NUMTs in analyses of human mtDNA. Current approaches include: (1) isolating mitochondria to enrich for mtDNA; (2) applying basic local alignment to identify NUMTs for subsequent filtering; (3) bioinformatic pipelines for NUMT detection; (4) k-mer-based NUMT detection; and (5) filtering candidate false positive variants by mtDNA copy number, VAF, or sequence quality score. Multiple approaches must be applied in order to effectively identify NUMTs in samples. Although next-generation sequencing is revolutionizing our understanding of heteroplasmic mtDNA, it also raises new challenges with the high prevalence and individual-specific NUMTs that need to be handled with care in studies of mitochondrial genetics.

## 1. Introduction

Nuclear-mitochondrial DNA segments (NUMTs) are fragments of the mitochondrial genome (mtDNA) that have been inserted into the nuclear genome of multiple organisms, including domestic cats, great apes, fruit flies, and humans [1,2,3,4,5]. NUMTs are likely due to multiple insertion events rather than duplications of nuclear DNA [6]. The concept of NUMTs was first suggested in 1967 [7] but experimental evidence of NUMTs only began appearing in the literature in the early 1980s [8,9]. Mitochondrial-like sequences in the nuclear genome have been identified in multiple species, including humans [10], yeast [11], mice [12], and others [13,14].

NUMTs have been used to define phylogenetic outgroups and elucidate mtDNA evolution [10,14]. Recent versus ancient nuclear insertions of mtDNA segments can be determined based on the degree of homology between NUMTs and the mtDNA equivalent within the individual. Once integrated into the nuclear genome, NUMTs undergo a slower mutation rate than the mtDNA, which is likely more similar to the slower mutation rate of the nuclear genome compared to the mtDNA [6,15,16]. Therefore, NUMTs that are dissimilar to their mtDNA counterparts are often thought of as “nuclear fossils”, fragments derived from ancient mtDNA [3,17]. In contrast, NUMTs that are more similar to their homologous mtDNA sequences are likely more recent insertions into the nuclear genome.

NUMTs can originate from any part of the mtDNA and are found throughout the nuclear genome [1,18]. The number of NUMTs found in the human genome varies, with most individuals harboring several NUMTs [1,3,18,19]. Based upon the characterization of NUMTs in more than 67,000 human genomes, NUMTs are estimated to arise de novo once in every 10^4^ births [1]. Comparisons of parent-child triads indicate that the incorporation of mtDNA fragments into the nuclear genome is an ongoing process [1]. NUMTs range in size from 24 bp to nearly the entire length of the mtDNA [1]. The majority of NUMTs are less than 500 bps in length and most frequently originate from the D-loop, a non-coding region containing many of the mtDNA regulatory elements [1].

NUMTs pose a challenge in studies evaluating mitochondrial genetic variation. Due to the high sequence similarity of NUMTs to the mtDNA within the individual, variants may be misidentified as being in the mtDNA rather than attributed to a NUMT. NUMTs interfere with the calculation of variant allele frequency (VAF), the number of copies of the mtDNA that contain a variant, used to define heteroplasmy [20]. Early studies reveal that NUMTs are often co-amplified with true mtDNAs, due to the use of non-specific primers in polymerase chain reaction (PCR) experiments or techniques that inadvertently select for nuclear DNA, creating additional challenges in sequencing techniques that involve the use of PCR [21,22,23].

NUMTs often do not encode functional proteins; however, some NUMTs are associated with disease [15]. One study mistook variants in NUMTs as missense variants in the mtDNA genes encoding subunits of cytochrome c oxidase and then associated those variants with Alzheimer’s disease [24]. The association of these NUMT-derived variants with Alzheimer’s disease has since been disproven, but this highlights the difficulties in attributing variants to the mtDNA versus NUMTs [23,25]. Further study of NUMTs is necessary to prevent the false identification of mtDNA variants. A number of methods are emerging for the improved detection of NUMTs. For this review, we will focus on the characterization of NUMTs and current methods for identifying NUMTs.

## 2. Transfer of mtDNA Segments into the Nuclear Genome

NUMTs are found throughout the entire human nuclear genome [1,15,26], but how mtDNA fragments get out of the mitochondrion and into the nucleus is not known. Currently, no consensus exists regarding the mechanism for the insertion of mtDNA segments into the nuclear genome. However, several studies have provided some insights into the potential mechanisms and non-random site selection of NUMT insertion.

The theory of mitochondrial endosymbiosis posits an evolutionary benefit of full-length mitochondrial gene transfer to specific loci in the eukaryotic nucleus that results in the production of a protein product [27,28,29]. The endosymbiotic relationship between the primitive cell and the bacteria from which mitochondria originate is thought to have occurred at the root of eukaryotic evolution, approximately 2.5 billion years ago [30], at which time much of the mtDNA was transferred to the nuclear genome, with the mitochondria retaining the genes encoding core catalytic subunits of the OXPHOS enzymes. However, NUMTs do not appear to be inserted as a functional transfer of mitochondrial genes to the nuclear genome, despite NUMTs of all lengths, from short fragments to the entire length of the mtDNA, occurring across different species [1,22]. While the mtDNA has been shown to be transferred to the nuclear genome in its entirety as a single NUMT in some individuals, often only fragments of the mtDNA are typically transferred, which are likely to be non-functional [1,3,18,19]. NUMTs may be inserted in a way that is similar or distinct from the process of full mitochondrial gene transfer to the nuclear genome. While full gene transfer of mitochondrial genes to the nuclear genome may result in the co-expression of both genomes to regulate mitochondrial function, NUMTs may serve a different function, or possibly no function at all.

NUMTs are found across all nuclear chromosomes as insertions; hence, a biased mechanism of insertion towards any given locus seems unlikely [1]. However, a bias for open chromosomal locations adjacent to A + T oligomers has been noted [31]. Nonetheless, NUMTs do not appear to be independent loci under the control of their own promoters/repressors. As such, NUMTs appear to be seemingly accidental inclusions of mtDNA in the nuclear genome without a functional presence.

NUMTs may be preferentially inserted at points of double-stranded DNA breaks. The insertion of mtDNA fragments at double-stranded DNA breaks in the nuclear genome is thought by some to be an intentional process occurring during non-homologous end joining, a major double-stranded DNA break repair process in eukaryotic cells. Non-homologous end joining, while an effective way to repair double-stranded DNA breaks, results in deletion errors, possibly resulting in disease-causing frameshifts [32]. Environmental stressors and events that create double-stranded DNA breaks, including ionizing radiation exposure [33] and age-related free radical generation [34], are associated with an increase in the insertion of NUMTs, termed numtogenesis. Interestingly, the inclusion of NUMTs at non-homologous end joining repair sites in the nuclear genome of yeast was associated with a 46% reduction in DNA deletions [35]. Hence, greater biological fitness may be conferred by the incorporation of NUMTs during non-homologous end joining compared to cells without insertions of NUMTs.

A key question in the field is how the mtDNA fragments come to be inside the nucleus and available for insertion into double-stranded DNA breaks. Some literature suggests the involvement of mitophagy in the creation of freely available mtDNA fragments for insertion as NUMTs [36]. Additionally, double-stranded mtDNA was found to be transported across both mitochondrial membranes through the voltage-dependent anion channel [37]. Whether or not this is the primary mechanism of mtDNA movement out of mitochondria to be available in the nucleus for insertion into the nuclear genome as a NUMT remains unclear.

## 3. Technical Approaches for Limiting NUMTs

**Figure 1:**
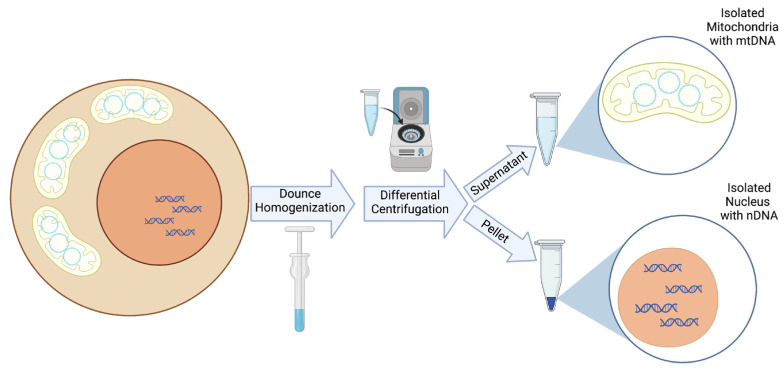
A schematic representation of mitochondrial and nuclear isolation. In brief, a membrane detergent is added to a pellet of whole cells or tissues. Next, dounce homogenization is performed with the specific number of dounce strokes dependent upon the cell or tissue type. Following homogenization, differential centrifugation over several steps first pellets nuclei and then mitochondria based on their different masses, allowing for the collection of both purified nuclear DNA (nDNA) and mtDNA from the same cells or tissue.

In addition to the computational approaches available for detecting NUMTs, several technical approaches can be used to limit the NUMTs in a sample. The most straightforward technical approach involves the isolation of mitochondria, thereby removing the nuclei, and hence, the nuclear genome and NUMTs, from the sample [38,39]. Mitochondrial isolation can be achieved with relative ease in cell pellets or whole animal or human tissue through the use of commercially available kits [40,41,42]. Differential centrifugation techniques employed by these kits result in minimal nuclear contamination, which would not be expected to contribute a sufficient number of reads during next-generation sequencing to be problematic in heteroplasmic mtDNA variant detection (Figure 1).

Following mitochondrial isolation, a method called Mito-SiPE (sequence-independent, PCR-free mitochondrial DNA enrichment) can be employed to determine the true mtDNA sequences and heteroplasmic variants present in the mtDNA sample. After mitochondrial fraction enrichment from cells or tissues, ultra-deep sequencing is performed to gain >80,000× coverage of the mtDNA, thereby limiting the introduction of false heteroplasmic variants from PCR enrichment or NUMT contamination [38]. Several PCR-based techniques such as Mito-tiling and rolling circle amplification [43] also enrich for mtDNA sequences, particularly in samples with a low mtDNA copy number; however, such approaches may also co-amplify NUMTs due to high sequence similarity.

## 4. Computational Identification of NUMTs

To prevent the interference of NUMTs in studies of mitochondrial genetic variation, some studies simply remove loci from existing catalogs of NUMTs from their analyses; however, this approach is likely not sufficient. Apart from common NUMTs that are widely shared, most NUMTs are rare and specific to individuals [1]. The use of bioinformatic approaches is required to efficiently and effectively identify all NUMTs in next-generation sequencing datasets prior to performing analyses of mtDNA variants with outcomes and phenotypes of interest.

**Figure 2:**
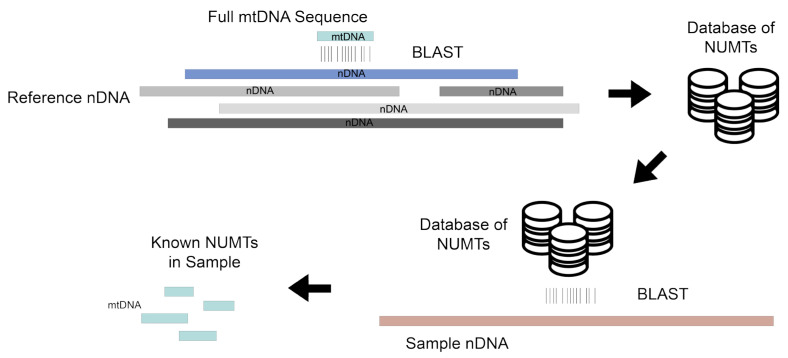
Using BLAST to identify NUMTs: The full mtDNA sequence from multiple individuals is aligned against the reference nuclear DNA (nDNA) with nucleotide BLAST in order to build a database of common NUMTs. Nucleotide BLAST searches performed between a sample nDNA and a database of common NUMTs can detect annotated ancestral NUMTs present in the sample nDNA, but cannot effectively detect polymorphic NUMTs specific to individuals, which are the majority of NUMTs found in humans. Hence, nucleotide BLAST will miss the vast majority of NUMTs, which are polymorphic NUMTs unique to individuals [1].

Before the advent of next-generation sequencing, NUMTs were primarily detected through a basic local alignment search (BLAST) of a PCR-amplified DNA sequence to reference genomes [20,44]. To discover novel NUMTs in a genome, exhaustive BLAST searches were also performed. By aligning the entire mtDNA sequence of multiple samples to the reference nuclear genome using a nucleotide BLAST, a database of common NUMTs in humans was built [45]. Similarly, an exhaustive BLAST search aligning a specific region of the mtDNA to the nuclear genome of different species provided additional insights into how NUMTs of certain mtDNA regions differ across species [46]. BLAST is still used to investigate or confirm whether annotated ancestral NUMTs are present in next-generation sequencing data, which can then be excluded prior to analyses of mtDNA variants with phenotypes of interest (Figure 2) [47]. However, more sophisticated tools are now available for NUMT detection in next-generation sequencing data and will be the focus of this section of the review.

**Figure 3:**
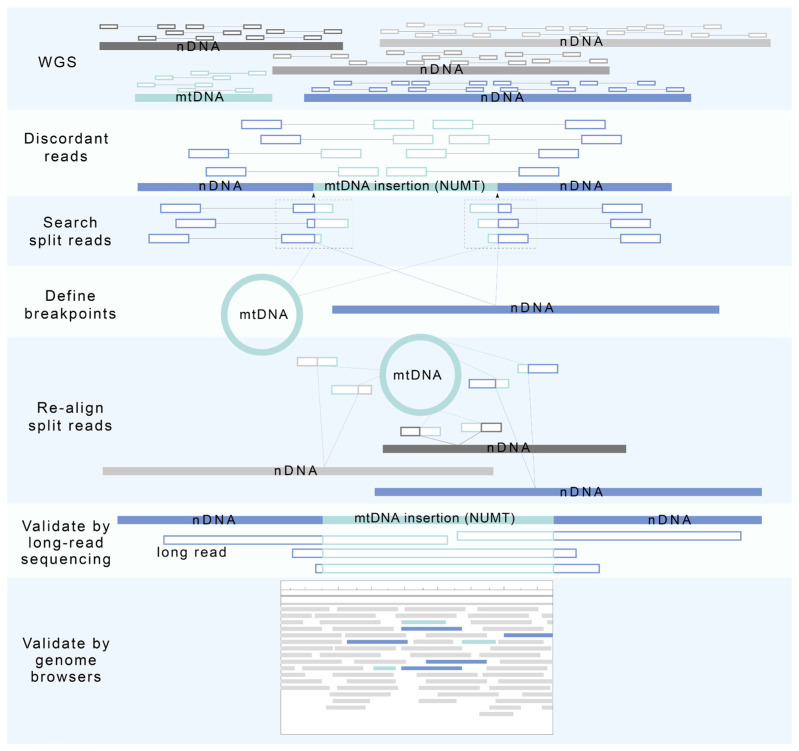
A novel NUMT detection pipeline for paired-end WGS data. In this pipeline, discordant read pairs, with one read in the pair aligning to the nuclear DNA (nDNA) and the other read aligning to the mtDNA, are first detected and clustered to locate a putative breakpoint. With multiple split reads retained, a breakpoint can be identified. Split reads are then re-aligned to the reference mtDNA and nDNA to locate the origin of NUMT insertion. Long-read sequencing and genome browsers can be used to further validate the location of detected NUMTs.

A novel NUMT discovery pipeline for paired-end whole genome sequencing (WGS) data has been developed and applied in multiple studies (Figure 3) [1,48,49]. In the pipeline, discordant paired-reads, with one read mapped to the nuclear genome and the other read mapped to the mtDNA, are selected and clustered based on their location, orientation, and insertion size. Discordant read clusters with more than five paired reads are used to identify the breakpoint location to determine the NUMT location within the nuclear genome. WGS reads in regions of putative breakpoints are then reviewed again in search of split reads, where half of the read is mapped to the nuclear genome, and half to the mitochondrial genome. With five or more split reads of good quality, the breakpoints can then be readily defined. To further locate the site of insertion of the NUMTs, split reads are realigned to both nuclear and mitochondrial reference genomes. Novel NUMTs in WGS data detected by this pipeline are further validated using long-read sequencing or through an additional examination on genome browsers.

**Figure 4:**
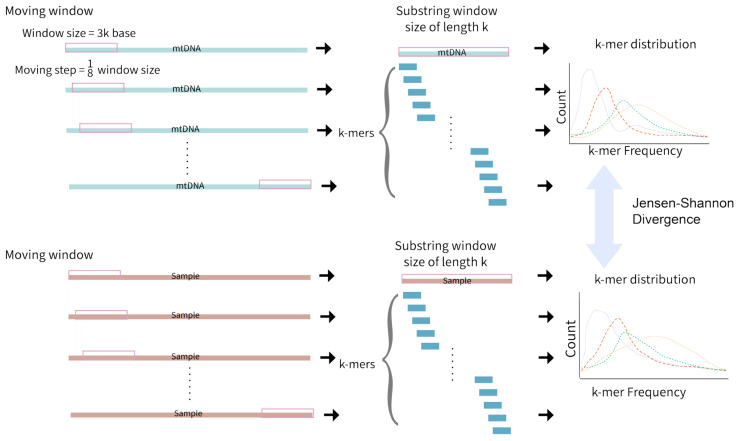
The k-mer-based NUMT detection method. A 3000 base pair moving window first takes a step of 1/8 window size on the full mtDNA sequence. Each window is sub-stringed with size k and the distribution of k-mers is recorded. The same moving window is applied to sample nuclear DNA (nDNA), and the k-mer distributions are compared with those from mtDNA using Jensen–Shannon Divergence. This approach cannot effectively detect polymorphic NUMTs specific to individuals when performed with reference genomes, and hence, may miss a number of NUMTs.

An alignment-free approach to detect NUMTs has also been developed (Figure 4). Local alignment assumes that the divergence between two sequences is small; hence, local alignments may fail to detect significant similarity between sequences with larger mutational dynamics, as is the case with NUMTs [50]. A k-mer-based NUMT detection method was developed that uses a moving window of 3000 bases. The algorithm moves a step of ⅛ the window size, targeting all NUMTs within the range of 3000 base pairs of length. After applying moving windows to both mtDNA and nuclear DNA in a sample, the frequency of k-mers in each window is recorded, resulting in multiple k-mer frequency distributions. Jensen–Shannon divergence is then used to measure the similarity between the two k-mer frequency distributions (one for each genome). A window size and moving steps of k = 7 was found to be optimal at efficiently providing unique k-mer distributions for ancestral NUMT detection [50].

**Figure 5:**
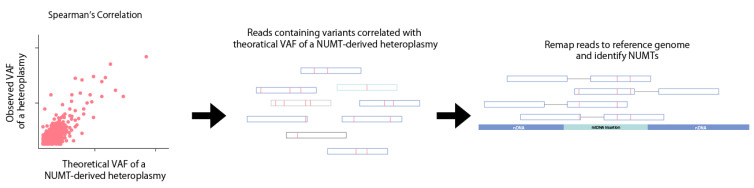
A NUMT detection method comparing the observed and theoretical VAFs of NUMT-derived heteroplasmic variants. Due to the relation of mtDNA coverage, nuclear DNA (nDNA) coverage, and mtDNA copy number, the theoretical VAF of a NUMT-derived heteroplasmic variant can be calculated. Heteroplasmic variants with a VAF that correlates with the theoretical VAF of a NUMT-derived heteroplasmic variant are likely false positives. The reads containing the false positive NUMT-derived heteroplasmic variant are then remapped to the reference genome to locate the NUMT insertion point.

NUMTs within WGS datasets can also be identified using mtDNA copy number (Figure 5). MtDNA copy number negatively correlates with NUMT-derived heteroplasmic mtDNA variants [51]. Most false positive NUMT-derived heteroplasmic variants have a VAF of 0–5% in human blood samples [51]. Theoretically, a NUMT-derived heteroplasmic variant will have a VAF of approximately 1/(1 + mtDNA copy number), where mtDNA coverage is twice the nuclear coverage. NUMTs can be identified by using Spearman’s correlation to identify variants with a VAF correlated with 1/(1 + mtDNA copy number), after which, the reads containing the candidate NUMT-derived variants are remapped back to the human reference genome. This method also identifies NUMT-derived heteroplasmic mtDNA variants where at least two variants are derived from the same NUMT insertion.

If the mtDNA copy number is high enough (>500 copies) for a sample, as is the case for highly energetic tissues, NUMTs are likely not problematic for detecting heteroplasmic variants with a low VAF of 1–5%. In tissues with high mtDNA copy numbers, most NUMT-derived heteroplasmic variants will have a VAF below the commonly used VAF thresholds of 3–10% for defining heteroplasmic variants [51]. However, most population-level studies use whole blood or peripheral blood mononuclear cells for WGS, which do not have sufficiently high mtDNA copy numbers to allow for false positive heteroplasmic variants to be confidently removed at low VAF thresholds. Hence, studies using WGS from blood samples require the implementation of additional computational strategies for NUMT detection.

To distinguish low-level heteroplasmic variants due to mtDNA variants rather than variants found in NUMTs, software such as DREEP [52] has been developed to provide DQS scores, a Phred-like quality score. DQS scores are measures of the deviation of the observed minor allele count from the expected error count, derived from a reference panel to indicate whether the mtDNA heteroplasmic variant is the result of a sequencing error. When mtDNA variants have a VAF ≥ 0.015 and a ≥4 DQS score, no false positive variants due to NUMTs are identified, suggesting that the combined thresholds for minor allele frequency and DQS is sufficient for high-confidence mtDNA variant identification [52]. Using a reference panel can be extremely problematic, however, as multiple studies have shown that samples have very different mtDNA copy numbers, even when the same DNA extraction kits and sequencing protocols are used [51]. As mtDNA copy number is closely related to the probability of identifying false positive variants due to NUMT contamination [51], using a reference panel like DREEP may be problematic.

To validate that the detected variants are truly from mtDNA, some bioinformatic pipelines generate a de novo mtDNA reference for each individual sample and conduct a second iteration of variant calling [43,53]. Using a de novo consensus mtDNA reference specific to each individual reduces potential bias introduced by the commonly used revised Cambridge Reference sequence [54], as more reads are aligned with high confidence using the de novo consensus mtDNA reference. The increase in the coverage afforded by the consensus reference results in higher confidence in the identification of variants. Although only applying a consensus mtDNA as a reference may fall short of minimizing the contamination of long NUMTs, the majority of the NUMTs are short insertions [1] and confirmation of the existence of long NUMT sequences necessitates the use of long-read sequencing. No studies have directly compared the mtDNA variant calling results between the revised Cambridge Reference sequence and the consensus reference sequence, but using a de novo consensus mtDNA reference for each sample will likely reduce the contamination of NUMTs, particularly in cell or tissue types with a low mtDNA copy number.

## 5. Conclusions

NUMTs appear in all humans and across the entirety of the nuclear genome. Emerging evidence suggests that there may be a physiological and possible protective role of NUMT insertion into nuclear double-stranded break regions. However, false positive heteroplasmic variants due to the presence of NUMTs remains a challenge in confidently identifying low-level heteroplasmic variants in mtDNA sequencing datasets. Nonetheless, the methods for minimizing and identifying NUMTs, both technical and computational, are rapidly advancing. Mitochondrial isolation with subsequent sequencing represents an ideal scenario for removing the effect of NUMT contamination in mtDNA sequences. When mitochondrial isolation is not technically feasible, employing multiple computational methods can aid in identifying and removing NUMTs to facilitate studies of heteroplasmic variants with low VAFs. Ultimately, it is critical to run one or more of the aforementioned countermeasures to ensure accurate heteroplasmic mtDNA variant detection in mitochondrial genome sequencing.

## Data Availability

Not Applicable.
